# Copy Number Variation in Familial Parkinson Disease

**DOI:** 10.1371/journal.pone.0020988

**Published:** 2011-08-02

**Authors:** Nathan Pankratz, Alexandra Dumitriu, Kurt N. Hetrick, Mei Sun, Jeanne C. Latourelle, Jemma B. Wilk, Cheryl Halter, Kimberly F. Doheny, James F. Gusella, William C. Nichols, Richard H. Myers, Tatiana Foroud, Anita L. DeStefano, the PSG–PROGENI and GenePD Investigators, Coordinators and Molecular Genetic Laboratories

**Affiliations:** 1 Department of Medical and Molecular Genetics, Indiana University School of Medicine, Indianapolis, Indiana, United States of America; 2 Department of Neurology, Boston University School of Medicine, Boston, Massachusetts, United States of America; 3 Center for Inherited Disease Research (CIDR), Institute of Genetic Medicine, Johns Hopkins University School of Medicine, Baltimore, Maryland, United States of America; 4 Center for Human Genetic Research, Massachusetts General Hospital, Boston, Massachusetts, United States of America; 5 Department of Neurology, Harvard Medical School, Boston, Massachusetts, United States of America; 6 Department of Genetics, Harvard Medical School, Boston, Massachusetts, United States of America; 7 Division of Human Genetics, Cincinnati Children's Hospital Medical Center, Cincinnati, Ohio, United States of America; 8 Department of Pediatrics, University of Cincinnati College of Medicine, Cincinnati, Ohio, United States of America; 9 Department of Biostatistics, Boston University School of Public Health, Boston, Massachusetts, United States of America; Public Library of Science, United Kingdom

## Abstract

Copy number variants (CNVs) are known to cause Mendelian forms of Parkinson disease (PD), most notably in *SNCA* and *PARK2*. *PARK2* has a recessive mode of inheritance; however, recent evidence demonstrates that a single CNV in *PARK2* (but not a single missense mutation) may increase risk for PD. We recently performed a genome-wide association study for PD that excluded individuals known to have either a *LRRK2* mutation or two *PARK2* mutations. Data from the Illumina370Duo arrays were re-clustered using only white individuals with high quality intensity data, and CNV calls were made using two algorithms, PennCNV and QuantiSNP. After quality assessment, the final sample included 816 cases and 856 controls. Results varied between the two CNV calling algorithms for many regions, including the *PARK2* locus (genome-wide p = 0.04 for PennCNV and p = 0.13 for QuantiSNP). However, there was consistent evidence with both algorithms for two novel genes, *USP32* and *DOCK5* (empirical, genome-wide p-values<0.001). *PARK2* CNVs tended to be larger, and all instances that were molecularly tested were validated. In contrast, the CNVs in both novel loci were smaller and failed to replicate using real-time PCR, MLPA, and gel electrophoresis. The *DOCK5* variation is more akin to a VNTR than a typical CNV and the association is likely caused by artifact due to DNA source. DNA for all the cases was derived from whole blood, while the DNA for all controls was derived from lymphoblast cell lines. The *USP32* locus contains many SNPs with low minor allele frequency leading to a loss of heterozygosity that may have been spuriously interpreted by the CNV calling algorithms as support for a deletion. Thus, only the CNVs within the *PARK2* locus could be molecularly validated and associated with PD susceptibility.

## Introduction

Copy number variants (CNVs), defined as structural changes in DNA consisting of deletions or duplications of segments larger than 1 kb compared to a reference genome [1], have been shown to be a disease producing mechanism in disorders such as Charcot–Marie–Tooth neuropathy, as well as a risk factor in some common, complex disorders such as schizophrenia and autism [2], [3], [4], [5]. The sizes of CNVs are quite variable. Some deletions and duplications span large physical distances encompassing several genes, while others span only a limited portion of a gene or no known gene at all.

Parkinson disease (PD) is the second most common neurodegenerative disorder affecting approximately 500,000 Americans. The genetic etiology of PD is complex, although mutations in five genes have been identified that lead to either an autosomal dominant or an autosomal recessive form of the disease [6]. Detailed analyses of these genes have identified a variety of disease producing mutations, including point mutations, exon duplications and deletions, as well as entire gene duplications and triplications. These findings suggest that CNVs, a term that includes exon or gene duplications or deletions, are an important disease mechanism contributing to PD pathogenesis.

Mutations in *PARK2*, the gene that encodes Parkin, are typically considered to act in an autosomal recessive fashion, with only those individuals having a mutation on both chromosomes developing PD. However, multiple reports have suggested that haploinsufficiency of *PARK2* may increase the risk of PD [7], [8], [9], [10]. We have recently shown that *PARK2* haploinsufficiency, specifically for a dosage mutation rather than a point mutation or small insertion/deletion, is a risk factor for familial PD and may be associated with earlier age of onset [11]. These data suggest a broader mechanism in which CNVs may play a role as a risk factor, increasing the likelihood an individual will develop PD.

In the present study, we replicate in an independent dataset the association of a single dosage mutation in *PARK2* with an increased risk of PD. This replication provides a positive control with which to compare different CNV filtering criteria as well as different genome-wide analysis strategies that can be used to explore the role of CNVs in a sample of familial PD cases and neurologically examined controls.

## Results

The final sample included 816 cases and 856 controls (Table 1). Controls tended to be younger than the cases and were more likely to be female. No control sample had a family history of PD. Whole blood was the DNA source for all of the cases, while lymphoblast cell lines (LCLs) was the DNA source for all controls.

**Table 1:** Sample demographics.

	PD Cases (n = 816)		Controls
	PROGENI	GenePD	NINDS Coriell Repository
	(n = 486)	(n = 330)	(n = 856)
Average age at onset (cases) or at enrollment (controls)	62.1±10.4	61.4±11.6	54.8±13.1
% Male	60.3%	57.8%	40.1%
% with parent reported to have PD	36.0%	23.9%	0%

**Figure 1:**
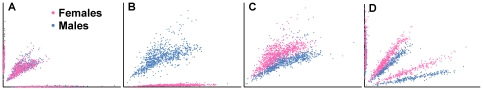
Scatter plots of raw probe intensities. A. A good marker, with three distinct clusters and males and females equally distributed in each cluster; B. Complete co-hybridization to sex chromosome, where all females are called as homozygotes and all males are called as heterozygotes; C. A monomorphic marker (i.e. a CNV probe) exhibiting partial co-hybridization to a sex chromosome, such that individuals cluster by gender, but mean Log R ratios do not differ by gender (p = 0.49); D. Polymorphic SNP exhibiting partial hybridization to a sex chromosome, where multiple distinct groups separated by gender.

The Illumina HumanCNV370 array provided intensity data for 370,404 probes. Illumina's BeadStudio software transformed the signal intensity data for these probes into Log R ratio (LRR) and B allele frequency (BAF) measures that could be used to generate CNV calls by PennCNV [12] and QuantiSNP [13]. Due to the limitations of these CNV calling algorithms, 1,357 markers (0.37%) within regions of known instability (telomeres, centromeres and immunoglobulin regions, boundaries previously delineated by Need et al. [4]) were excluded from analysis. An additional 1,165 autosomal markers (0.31%) demonstrated a gender-specific pattern indicative of hybridization to a sex chromosome in addition to, or instead of, the target locus (see Figure 1) and were similarly removed. CNV calls generated when including gender-linked markers were compared to those calls generated when excluding such markers. Exclusion of these markers resulted in the removal of approximately 1% of identified CNVs, which were presumably spurious. New deletions and duplications were also called (an additional 1% of calls) after excluding the gender-linked markers, indicating that some of these excluded markers had shown evidence of copy number equal to two. By removing gender-linked markers that were presumably showing evidence of two copies, these new deletions and duplications were then called. The final number of markers used for CNV detection was 367,882.

**Figure 2:**
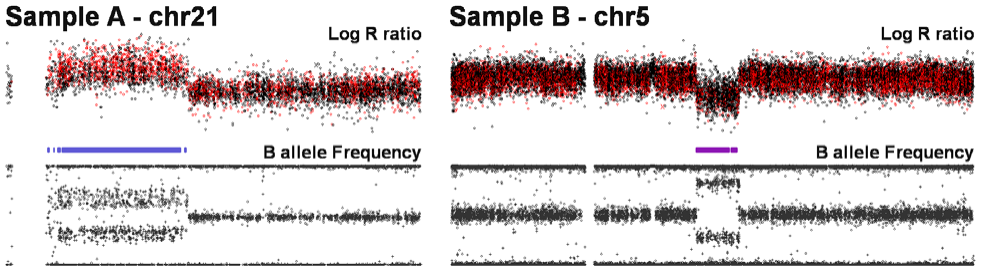
Plots of large chromosomal rearrangements. Sample A harbors a large duplication with the region indicated by a blue bar (average Log R ratio is increased, and B allele frequency (BAF; proportion of alleles estimated to be the B allele) match the 4 expected proportions of 0.0 = AAA, 0.33 = AAB, 0.66 = ABB, 1.0 = BBB). Sample B harbors a large deletion with the region indicated by a purple bar (decreased Log R ratio, with no heterozygotes (BAF = 0.50)); however, since BAF are not limited to values of 0 and 1, the deletion appears to be mosaic.

**Figure 3:**
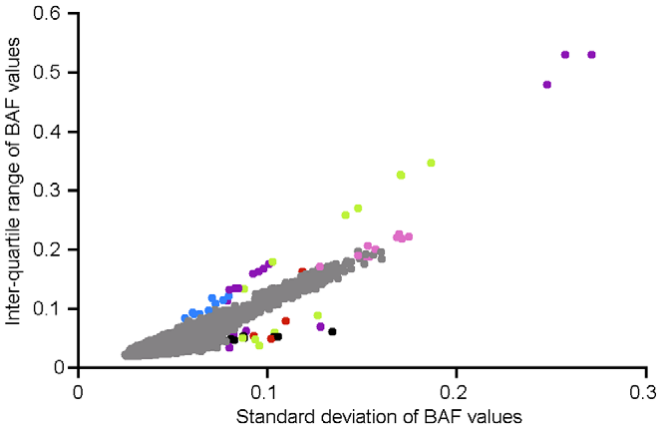
Detecting mosaicism. Gray dot = normal Log R ratio (LRR) and B allele frequency (BAF) distributions; Green dot = mosaic pattern with a significant enough deviation in mean LRR to be called as a CNV – such chromosomal arms were removed from analyses; Purple dot = mosaic pattern without significant LRR deviation – the CNV calling algorithms did not call these as CNVs; Blue dot = faint mosaics – not called; Red dot = multiple distinct mosaicism events – chromosomal arms removed from analyses; Black dot = normal LRR and BAF distributions – the reason they are outliers is unknown; Pink dot = no mosaicism pattern, but very noisy – all of these samples had already been flagged as having unacceptably high LRR standard deviations and had already been removed from analyses.

Whole chromosomal arm mosaicism was detected by analyzing the BAF distribution of each chromosomal arm of each individual. There were 44 chromosomal arms from 35 samples (13 cases, 22 controls) that were flagged as outliers and demonstrated evidence of mosaicism (Figures 2 and 3), generally for a majority of the length of the chromosomal arm. While these regions had distinct BAF patterns indicating mosaicism (i.e. deletions with BAF values >0 and BAF values <1), LRR values were not always significantly increased or decreased, in which case the CNV calling algorithm did not flag the region as a CNV. Those that were not called tended to have a lower percentage of mosaicism (BAF heterozygote bands closer together) and generally included only one breakpoint (i.e. encompassed the telomere). Those mosaics in which CNVs were called, frequently had two breakpoints (i.e. did not encompass a telomere), yielding large chromosomal rearrangements, similar to those seen in recent reports on schizophrenia [4], [5]. These included 3 PD cases with deletions (chr2q/6Mb/onset = 50, chr5q/11Mb/onset = 72, chr6q/7Mb/onset = 75) and 1 PD case with a duplication (chr21q/10Mb/onset = 30). All four of these PD cases showed evidence of mosaicism (see Figure 2). All mosaics called as CNVs by PennCNV or QuantiSNP were excluded from analyses (7 cases, 9 controls; 19 arms; 3 controls had two mosaic arms).

Previously, an increased risk of PD was found for those having a single *PARK2* dosage mutation [11]. We attempted to replicate this association using only those samples that are independent of the discovery set of Pankratz et al. [11]. The replication set used in our current analyses included 66 PROGENI cases, all 330 GenePD cases and the 846 Coriell controls. We excluded three individuals harboring *PARK2* CNVs on both alleles (compound heterozygotes). Single CNVs were identified in 10 of the 396 independent cases and 8 of the 856 controls, yielding an odds ratio of 2.7 (p = 0.03).

Of the ten cases in which CNVs were identified, two had already been molecularly tested for *PARK2* mutations and found to be heterozygous for an exonic deletion [10]. The two samples molecularly tested were from the GenePD study and were independent from the discovery set. Combined with the seven PROGENI samples from the current GWAS study that overlapped with the previous study of *PARK2* haploinsufficiency [11], a total of nine out of nine molecularly tested samples (100%) replicated with regard to copy number (deletion versus duplication). The breakpoints were not always precise, which was presumably due to variation in LRR values and the relatively sparse marker set. For example, a duplication of exons 5–8 that was verified using both MLPA and quantitative PCR was called as a duplication of exons 4–6 by PennCNV. A deletion defined molecularly as spanning only exon 2 was called as spanning both exons 2 and 3 by PennCNV. Of note, two mutations that included exon 2 were called by PennCNV as being restricted to intron 2.

PennCNV called 22,685 CNVs that had a confidence value of 10 or higher and that spanned at least 5 SNPs. Of these, 2,195 (9.8%) met the Conservative criteria (≥20 probes, ≥100 kb) and 20,073 (88.5%) met the Common criteria (≥5 probes and contain at least one SNP/CNV probe that was observed to be deleted or duplicated in our dataset 3 or more times). The intersection of the two filters contained 1,883 CNVs and the union contained 20,385 CNVs. There were 312 calls meeting the Conservative criteria that were restricted to regions where only one or two individuals contained variants and thus failed the Common criteria. There were 2,300 CNV calls (10.1%) that did not meet the criteria for either the Conservative or Common approach and were not analyzed in the union set. Of these, 1,568 contained 5–9 markers (avg. size 36 kb), 498 contained 10–14 markers (avg. size 71 kb), 199 contained 15–19 markers (avg. size 110 kb), and 35 with more than 20 markers (but less than 100 kb; avg. size 79 kb). The Gene-centric approach, which takes the union of the two other approaches and identifies the subset that overlaps a portion of at least one RefSeq gene, contained 8,746 CNVs or 42.9% of those possible.

Genome-wide analyses of locus-specific CNV associations were performed using CNV calls from two different algorithms (PennCNV and QuantiSNP) and using two different methods (position and 400 kb windows). Multiple comparisons were corrected within each analysis via permutation testing. To control for multiple testing across approaches, a conservative (given the correlation between the various permutations) Bonferroni correction of 0.0125 was applied to reach study-wide significance.

PennCNV showed a trend at the *PARK2* locus (p = 0.04; Figure S1A) that was not significant after correcting for multiple tests. Only when analyzed using the Conservative criteria (CNVs spanning at least 100 kb and 20 markers) and 400 kb windows was the finding genome-wide significant (p = 0.007; see Table S1). QuantiSNP did not show strong evidence of association for *PARK2* using any filtering criteria (p = 0.08–0.16). Other regions were similarly discordant between the two calling algorithms (chr1q, chr4q, chr5q, chr8p; see Table 2). Only two regions yielded consistent, genome-wide, and study-wide significant findings (p<0.0001). Each region contained a single gene: *DOCK5* and *USP32* (see Table 2). The CNVs in *USP32* were exclusively single deletions (copy number equal to 1) and were identified in 30 cases and 8 controls, yielding an odds ratio of 4.0 (Figure S1B). In contrast, the CNVs in *DOCK5* were quite common and included both deletions and duplications, with copy numbers ranging between 1 and 3 (Figure S1C). Upon further examination, all CNV calls in *DOCK5* overlapped the same 6 monomorphic CNV probes in intron 1. When the chromosome was reanalyzed without these 6 probes, the minority of CNV calls that extended beyond these markers were not called.

**Table 2:** Results (p-values) for regions with an empirical genome-wide p-value<0.20 for any test.

			PennCNV		QuantiSNP	
Location	Gene	Test^1^	Union	Gene-centric	Union	Gene-centric
chr1:173049146–173078950	85 kb from	P	0.17	0.10	1.00	1.00
	*PAPPA2*	W	0.13	0.07	1.00	1.00
chr4:71528873–71716513	overlapping	P	1.00	1.00	0.17	0.12
	*ENAM*	W	1.00	1.00	0.02	0.01
chr5:151389412–151513092	105 kb from	P	0.02	1.00	0.65	1.00
	*GLRA1*	W	0.02	1.00	0.39	1.00
chr6:162471089–162677104	within	P	0.83	0.64	0.91	0.79
	*PARK2*	W	0.04	0.02	0.13	0.08
chr8:7575048–7575048	gene	P	1.00	1.00	1.00	1.00
	desert	W	0.23	0.13	1.00	0.99
chr8:25038472–25171648	within	P	**0.0001**	**0.00006**	**0.0001**	**0.0001**
	*DOCK5*	W	**0.0001**	**0.0001**	**0.0001**	**0.0001**
chr11:84948993–84958207	within	P	1.00	1.00	0.04	0.03
	*DLG2*	W	0.20	0.11	0.02	0.009
chr17:55581582–55809920	within	P	**0.0006**	**0.0005**	**0.0007**	**0.0002**
	*USP32*	W	**0.0006**	**0.0005**	**0.001**	**0.0003**

Those p-values in **bold** are significant both genome-wide and study-wide.

1 Tests were either based on a specific position (P) or based on a 400 kb Window (W).

All CNV calls for the *USP32* locus overlapped either intron 2 or exon 2 (see Figure S1B). We therefore designed sets of probes to capture each of these regions and repeated each region twice to molecularly validate the CNV calls using real time PCR. In replicate 1, nine cases with PennCNV deletion calls were compared to two cases without deletions calls. In replicate 2, the same nine cases were compared to two cases that not only lacked deletion calls, but had LRR values near zero across all markers in the region. In each set of results, the quantity of DNA for each sample was compared to the mean quantity of the two controls. Proportions less than 0.80 were flagged as possible deletions and proportions above 1.20 were flagged as possible duplications. Results were consistent between the two replicates, with one case yielding a proportion below the lower limit (mean proportion across replicates = 0.74 for both probes) and one case yielding a proportion above the upper limit (mean proportion = 1.57 for both probes). Therefore, 89% of the *USP32* deletions calls failed to replicate molecularly using real time PCR.

**Figure 4:**
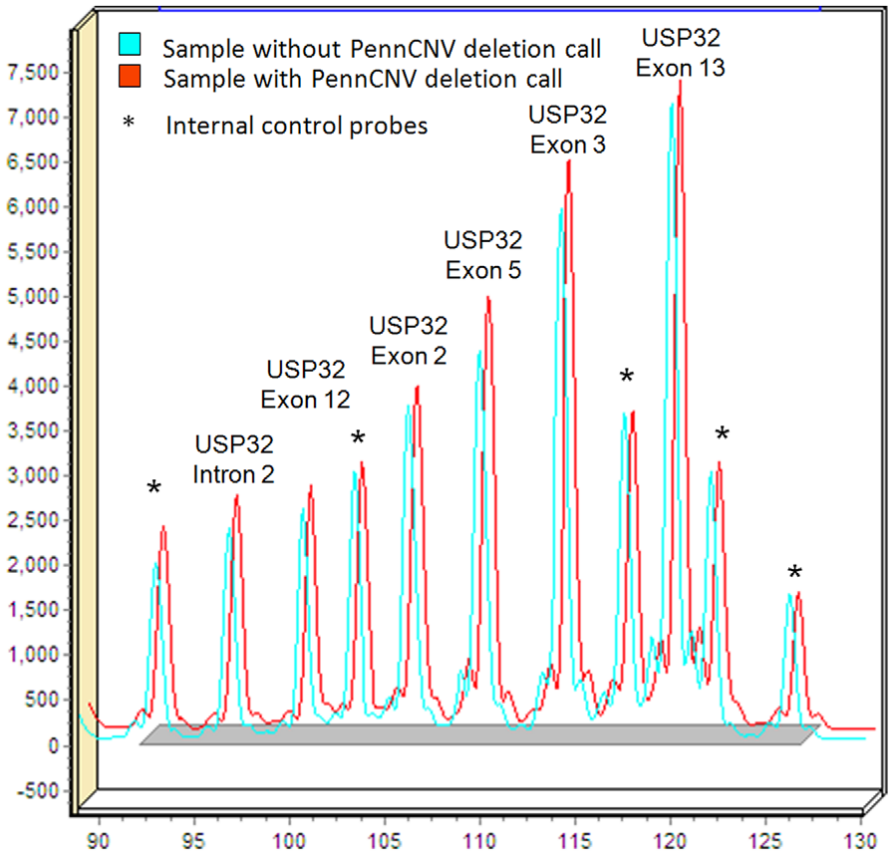
MLPA of *USP32* exons. A two color overlay shows a representation of the capillary electrophoresis peak profiles from one sample with a PennCNV deletion call (shown in red) and one sample without a PennCNV deletion call (shown green) in the *USP32* region. Internal control probes are also indicated. Every probe in the sample presented a normal amplification pattern, suggesting normal dosage for both copies of *USP32*.

We also used MLPA as an alternate validation method. Six samples with and fifteen samples without a PennCNV deletion call in the *USP32* gene (Figure 4) were investigated. No deletion was detected in any tested sample. All samples yielded similar results consistent with a copy number of two at all probes targeting the *USP32* gene. The probes on the X chromosome showed one copy in male individuals, indicating that the assay worked appropriately.

**Figure 5:**
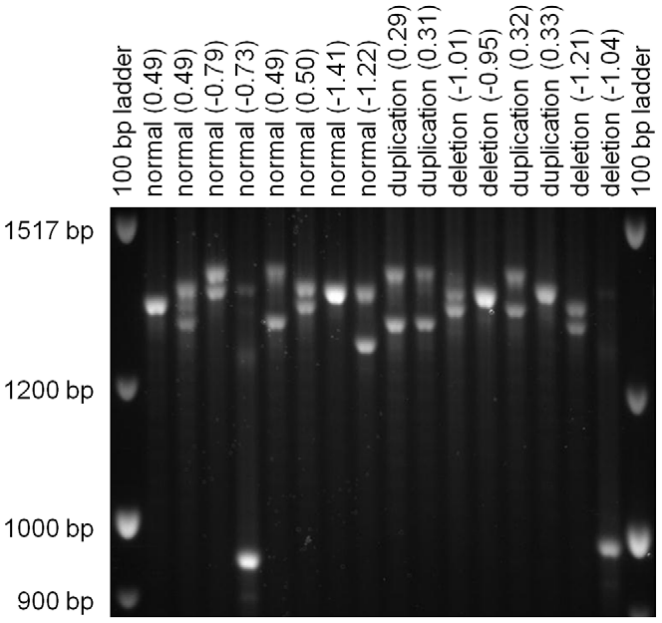
Gel electrophoresis of DOCK5 VNTR. This image of 16 samples is representative of all 95 samples run. PennCNV calls are listed for each sample (normal, deletion, duplication). The mean Log R ratio for the 6 monomorphic CNV probes in *DOCK5* is listed in parentheses. A few lanes showed evidence of a third band, assumed to be heteroduplexes of the two alleles. Estimated number of copies of the 32 bp repeat did not correlate with PennCNV calls or with mean Log R ratio. The finding is likely a result of artifact due to DNA source (blood versus LCLs).

Analysis of the underlying sequence of the *DOCK5* region (See Figure S2) and of the primer sequences of the 6 probes consistently deleted and duplicated revealed that the probes capture variable numbers of a tandem repeat (VNTR) and not the larger deletions and duplications that are normally included in the definition of a CNV. The 32-base pair repeats are found in three forms that vary at only two base pair positions. The reference genome contains 13 copies of form A, 2 copies of form B, and 13 copies of form C (See Figure S2). Three of these probes are nested, redundant probes for form A, and the others are nested, redundant probes for form C. The region containing the VNTR were amplified and separated by size using gel electrophoresis. Summed allele size (estimated number of 32 bp repeats for each allele added together) was not significantly different between samples with a PennCNV deletion call and a PennCNV duplication call (p = 0.69) or with mean LRR across the 6 monomorphic *DOCK5* probes (p = 0.99). A shorter allele with a size around 960 bp (see Figure 5) was noted in 8 subjects; however, this allele was seen in individuals without a PennCNV deletion call and was seen in equal frequencies in the 52 cases and 43 controls that were genotyped.

## Discussion

Despite the increase in the study of CNVs as a potential disease risk factor, there is still no consensus on the best approach for the detection or analysis of CNVs. A prior genome-wide study of CNV in Parkinson disease using a relatively small sample (273 cases and 275 controls) and visual inspection of LRR and BAF to identify CNVs, found CNVs within *PARK2* in both cases and controls. A single locus for which CNVs were identified in multiple cases but not in controls could not be validated by molecular analysis [14].

Here, we present the results of the first systematic genome-wide analysis of CNVs for PD using CNV calling algorithms. We replicated the association of PD susceptibility with *PARK2* CNVs in an independent sample. In addition, we detected CNVs in two novel genes, *DOCK5* and *USP32*, associated with an increase in risk for PD at genome-wide significance. However, neither of these novel loci could be validated with independent molecular tests.

The *DOCK5* probes capture a VNTR, and all of the current Illumina arrays with enhanced copy number probes (370-Duo, 660W-Quad, 1M-Duo, Omni) have the same six CNV probes for this region. A recent study of CNVs in the Wellcome Trust Case Control Consortium datasets found artifacts between samples with DNA isolated from LCLs versus samples isolated from whole blood [15], particularly at probes tagging regions with known VNTRs. Because all cases in this study were obtained from whole blood and all controls were from LCLs, this is likely the reason for the spurious association at the *DOCK5* region.

The finding of *USP32* (encoding Ubiquitin-specific protease 32) was particularly intriguing because it plays a role in the ubiquitin proteasome system (UPS) that also includes the gene product of *PARK2* (Parkin). However, the *USP32* finding also failed to replicate using either real-time PCR or MLPA. Visualizing the LRR and BAF values for these regions revealed that most individuals (with or without a PennCNV call) had BAF values exclusively at 0 and 1 across this region, which is consistent with a deletion call. However, this phenomenon was due to a series of nine SNPs with low minor allele frequencies (MAF<0.05) that were in tight LD with one another (r^2^>0.75). Since both PennCNV and QuantiSNP use BAF values in their calculations (without comparing allele frequencies or BAF distributions across individuals), this is one possible explanation why so many deletions were called in this region, as the frequency of the haplotype containing the minor allele of all nine SNPs was 0% in those assigned a deletion call in *USP32*, compared to 4.1% in the rest of the sample. However, while the frequency was lower in cases (3.9%) than it was in controls (4.2%), the difference was not significant and thus not sufficient to explain why CNV calls were made more often for cases than for controls. It is possible that DNA source may have played a role at this locus as well.

Duplications and triplications of *SNCA* are a well-documented cause of PD [6]. One case in the present study was identified as harboring a 3 Mb duplication that contained *SNCA* as well as 36 other genes (age of onset = 44). One additional case (age of onset = 69) was found to be mosaic for a duplication containing 80% of chromosome 4q (including *SNCA*), and was therefore excluded from analyses. However, since it is unknown if this mosaicism is present in the relevant brain regions, we cannot infer whether or not this duplication is disease causing. No CNVs were observed in or around *PARK6* (*PINK1*), *PARK7* (*DJ1*), *PARK8* (*LRRK2*) or *PARK9* (*ATP13A2*).

Both of the CNV calling algorithms generated CNVs in *USP32* and *DOCK5* that were significantly associated with disease. However, across the genome QuantiSNP, on average, generated both a higher number of CNVs per person and larger CNVs per person. Upon manual inspection of LRR plots, QuantiSNP would frequently call deletions in locations where a small proportion of markers actually had LRR values above 0, which would normally indicate a copy number of two, trending towards a copy number of three. This indicates a lower threshold for calling a CNV and would explain both the higher frequency and the longer average length of the CNV calls. While this may lead to fewer false negatives, it may also lead to more false positives. Conservative CNV calls in the *PARK2* region were significantly associated with disease when called by PennCNV (genome-wide empirical p = 0.007) but not when called by QuantiSNP (p = 0.16).

As described above, multiple filters were used to improve the quality of CNV calls. In retrospect, the Conservative approach (>100 kb and ≥20 markers) had the lowest false positive rate, since it did not flag *DOCK5* or *USP32*, and the highest power to detect *PARK2*, a true positive, at genome-wide significance. However, no new CNV associations were detected with the Conservative criteria. Aside from *PARK2*, the smallest nominal p-value observed using the Conservative filter was *MACROD2* (nominal p = 0.047; genome-wide p = 0.98; see Text S1 for more detail). The Conservative approach also fails to detect nearly all of the known copy number polymorphisms, which tend to be much smaller. It also ignores most of the monomorphic probes specifically added to the 370 Duo and more recent chips to identify copy number variation, since very few of these regions are tagged by more than 20 of these markers. Therefore, we also considered CNVs that overlapped a marker that was reported as deleted or duplicated three or more times.

Strengths of this study include the exclusive use of familial PD, which is likely to have a greater genetic contribution and, therefore, greater power to detect association than idiopathic PD. In addition, careful quality assessment was performed for the samples analyzed for CNVs, the markers used in the CNV calling algorithms, and the filters applied to the CNVs that were called. Limitations of the study include the relatively sparse marker set on the Illumina 370Duo compared to newer arrays and the stratification of DNA source by affection status.

In summary, we have detected association of PD with CNVs in *PARK2* at genome-wide significance, but failed to detect any additional loci that could be molecularly validated. Our experience indicates that CNV calls spanning only 5 SNPs should be met with skepticism. Furthermore, researchers should be wary of CNV probes meant to tag VNTRs (as opposed to traditional deletions and duplications), especially if cases and controls come from different DNA sources. Finally, more work needs to be done to investigate whether regions with several markers with low minor allele frequency may lead to spurious deletions calls in algorithms such as PennCNV and QuantiSNP.

## Methods

### Sample and Genotyping

A genome-wide case control association design was employed to identify genes contributing to PD susceptibility [16]. All PD cases had a positive family history of disease and were ascertained as part of two ongoing studies of familial PD: PROGENI and GenePD (see Table 1 for demographic information). All cases completed a uniform neurological evaluation that employed PD diagnostic criteria based upon a modified version of the United Kingdom PD Society Brain Bank Criteria [17]. Whole blood was the DNA source for all PD cases. Control samples were obtained from LCLs from the NINDS Human Genetics Resource Center DNA and Cell Line Repository (Camden, NJ). All control samples were reported to be white, non-Hispanic. Appropriate written informed consent was obtained for all samples included in this study. This study was approved by both the Institutional Review Board of Boston University Medical Campus and the Indiana University Institutional Review Board.

Genotyping was performed by the Center for Inherited Disease Research (CIDR) using the Illumina HumanCNV370 version1_C BeadChips (Illumina, San Diego, CA, USA) and the Illumina Infinium II assay protocol [18]. Intensity data were collected for 23,573 probes specifically designed to detect copy number variation. Detailed review of the data was performed to assess the quality of the samples [16]. All samples analyzed in the GWAS were considered for inclusion in the CNV analyses (857 familial PD cases and 867 controls). Those samples with high quality intensity data were used as the reference samples when the data was reclustered (see Text S1 for more detail).

All cases were known to be negative for the *LRRK2* G2019S mutation, and many, but not all, were also screened for mutations in *PARK1* (*SNCA*; n = 702 screened), *PARK2* (*parkin*; n = 593), *PARK7* (*DJ1*; n = 328), and *NR4A2* (*Nurr1*; n = 550) [9], [10], [11], [19], [20], [21], [22], [23], [24]. All PD cases with a *LRRK2* G2019S mutation as well as those known to have a mutation in both of their copies of *PARK2* were excluded as potential cases, since these mutations were believed to be sufficient to cause disease. However, PD cases with a mutation in only one of their two *PARK2* genes were included, since there was not definitive evidence that a single *PARK2* mutation was sufficient to cause disease. No mutations were found in those screened for *SNCA*, *DJ1*, or *NR4A2*.

### Marker filtering criteria

Due to the limitations of CNV calling algorithms, markers within regions of known instability (telomeres, centromeres and immunoglobulin regions, boundaries previously delineated by Need et al. [4]) were excluded from analysis. In addition, multiple criteria were used to identify markers demonstrating evidence of hybridization to chromosomal regions other than the target locus (see Figure 1B–D). Predicting gender using allele frequency differences or mean LRR values were helpful, but not sufficient in identifying all gender linked makers. Ultimately, three logistic regression models were used to predict gender. Each regression model contained two independent variables, which were found to be more sensitive than testing each of the variables separately (diagonal separation in two-dimensional space). The three sets of two independent variables for these models were: 1) normalized probe intensity pairs, X and Y, 2) R and Theta, and 3) BAF and LRR. Markers were removed if the minimum p-value, for any of the six variables considered, across the three models exceeded genome-wide significance (p-value<2.2×10^−8^; based on 370,404 markers and 6 variables). All gender-linked markers were removed from the final analyses.

### Detection of whole chromosomal arm mosaicism

CNVs that span the entire arm of a chromosome have been detected. Typically, these are the result of somatic loss or gain and often exhibit a mosaic pattern, with some cells containing a normal karyotype. The loss of an entire chromosomal arm is frequently an artifact of the lymphoblast immortalization process, which is relevant since all controls were from LCLs, and all the cases were from whole blood; however, in our sample, mosaicism was frequently seen in DNA derived from whole blood. To detect whole chromosomal arm mosaicism, each arm of each chromosome of each individual was analyzed separately. As described in Text S1, true germ line deletions will exhibit B alleles only near 0 and 1 (two bands) and duplications will yield four equidistant bands. If a sample exhibits a mosaic pattern, then a deletion will yield four bands (See Figure 2B), and a duplication will have the middle two bands closer to the midline. Chromosomal arms demonstrating evidence of mosaicism were identified by taking all BAF values between 0.15 and 0.85 and plotting the standard deviation of these BAF values on the X axis and the interquartile range (IQR) of these same values on the Y axis (Figure 3). Outliers were inspected visually by plotting all LRR and BAF values for the chromosome of the individual in question (as seen in Figures 2A and 2B). To eliminate possible bias, all samples demonstrating evidence of mosaicism for called CNVs were removed from further analysis (n = 16).

### CNV calling algorithms

There is currently no consensus regarding the best algorithm to call CNVs. Therefore, two frequently used CNV calling algorithms were employed, PennCNV [12] and QuantiSNP [13], and we have compared results from these two algorithms in our dataset. Commonly used parameters and thresholds for these programs were used to filter the samples down to the final dataset of 816 cases and 856 controls (see Text S1 for more detail).

### CNV filtering criteria

Two complementary filtering approaches were applied to minimize false positive CNV calls. The first approach, which we refer to as Conservative, focused on large CNVs that were greater than 100 kb and spanned at least 20 markers. Similar to previous studies [4], we also employed a second approach, which focused on common CNVs (Common approach). Less stringent criteria were applied for this Common approach, because, by definition, they occur at loci already identified as copy number variable regions or appear in multiple individuals within a study. Common CNVs were required to span at least 5 SNPs and to contain at least one SNP/CNV probe that was observed to be deleted or duplicated in our dataset 3 or more times. We analyzed the union of these two sets filtering criteria (see Text S1 for a comparison of the results of each filtering criteria).

We then performed secondary analyses that limited our analyses to those CNVs that overlapped a portion of at least one RefSeq gene (Gene-centric approach). We did not require the CNVs to specifically overlap an exon, since deletions molecularly confirmed to span an exon could be called by PennCNV as having boundaries that are exclusively intronic due to the relatively sparse marker set employed in the current study.

### Replication of the PARK2 locus

Those samples that did not overlap with the previous study of *PARK2* haploinsufficiency [11] were included in a replication sample. This included 66 cases from the PROGENI study, 330 cases from the GenePD study, and all 856 controls. CNVs that passed all filtering criteria and overlapped any portion of *PARK2* (chr6:161,688,579–163,068,824) were considered (Gene-centric CNV definition). Those individuals found to have two hitherto unknown *PARK2* mutations (compound heterozygotes) were not included in the analysis (n = 3). Fisher's exact test, using 1 df, was used to determine if cases were more likely than controls to harbor a single *PARK2* CNV. While samples that overlapped with the prior study of haploinsufficiency were not included in the *PARK2* replication analysis, they were used to verify that the CNV calling algorithms were able to properly call a molecularly validated CNV.

### Genome-wide association strategies

To test the hypothesis that particular CNVs would be found at increased frequency in PD cases as compared with controls (one-sided Fisher's exact test with significance determined via permutation), we performed two analyses using PLINK [25]. We first tested genome-wide whether the presence of a CNV at a particular position was found more frequently in cases versus controls. We then defined 400 kb windows across the genome (200 kb up- and down-stream from every marker) and tested whether the total number of CNVs within a given window was more common in PD cases than in controls. These tests were performed for each of the CNV definitions and empirical, genome-wide p-values were generated by permuting affection status.

### Molecular Validation

We sought to molecularly validate large statistically significant deletions and duplications identified in *USP32* and *PARK2* using both real-time PCR and multiplex ligation-dependent probe amplification (MLPA). Applied Biosystems' Assay by Design service was used to design fam-labeled TaqMan gene expression assays for targeted regions. Genomic DNA samples were quantitated by Pico Green fluorescence in triplicate with the Quant-iT PicoGreen dsDNA Kit (Molecular Probes, Eugene, OR). After quantitation, 50 ng of genomic DNA was used in a real-time absolute quantitation assay for the region in question, performed on the 7300 Real Time PCR System (Applied Biosystems) as previously described [11].

We used MLPA as a second approach to molecularly validate the inferred large CNVs. A custom assay with 11 probes was designed to capture exons 2, 3, 5, 12, and 13 of *USP32* and intron 2 of *USP32*, as well as probes on other chromosomes to serve as controls. The MLPA assay was performed as described previously [10], [11]. Peak height and area were then compared between samples with and without a PennCNV deletion call. Values between 0.8 and 1.2 were considered normal.

We also sought to molecularly validate the length of the 32 bp repeats identified in *DOCK5*. PCR products obtained by amplification using primers designed to flank the CNV (sequences available on request) were molecularly assessed by gel electrophoresis. After electrophoresis through 1% agarose in 1× TBE, products were visualized by ethidium bromide staining and UV light. Gels were run with a 100 bp ladder at a low voltage (40 V) for an extended period (24 hours) to ensure optimal separation of the PCR products and then photographed. The resulting PCR products were sized by comparing the coordinates of the pixel at the center of the PCR band and coordinates of the two nearest bands on the 100 bp ladder. Length (in base pairs) was assigned in proportion to the distance to the vertical positions of these reference bands. The number of 32-mer repeats was then calculated by subtracting the number of base pairs between the primers and the repeated sequence and dividing by 32. Summed allele size was then compared between samples with deletion calls and duplication calls using Student's t-test.

## Supporting Information

Figure S1 **Visualization of CNVs within** ***PARK2*** **(A),** ***USP32*** **(B), and** ***DOCK5*** **(C).** **Red** = Deletion in a case; **Pink** = Duplication in a case; **Dark green** = Deletion in a control; **Light Green** = Duplication in a control; **Red** followed by **×10** means that ten cases harbored a deletion with the exact same breakpoints; all CNVs displayed in Panel C overlap the same six monomorphic CNV probes (the smallest CNV, deleted ×70 in cases and ×36 in controls); the small arrows in the gene figure indicate direction, the large arrows indicate that not all of the gene is displayed, and the bars indicate exons.
(TIF)

Figure S2 **Sequence for the** ***DOCK5*** **region and for the primers for the probes in that region.**
(DOC)

Table S1 Comparison of genome-wide results across CNV filters for regions with an empirical genome-wide p-value <0.20 for any test.
(DOC)

Text S1 **Additional Methods and Results.**
(DOC)

## References

[1] Feuk L, Carson AR, Scherer SW (2006). Structural variation in the human genome.. Nat Rev Genet.

[2] Sebat J, Lakshmi B, Malhotra D, Troge J, Lese-Martin C (2007). Strong association of de novo copy number mutations with autism.. Science.

[3] Stefansson H, Ophoff RA, Steinberg S, Andreassen OA, Cichon S (2009). Common variants conferring risk of schizophrenia.. Nature.

[4] Need AC, Ge D, Weale ME, Maia J, Feng S (2009). A genome-wide investigation of SNPs and CNVs in schizophrenia.. PLoS Genet.

[5] Walsh T, McClellan JM, McCarthy SE, Addington AM, Pierce SB (2008). Rare structural variants disrupt multiple genes in neurodevelopmental pathways in schizophrenia.. Science.

[6] Pankratz N, Foroud T (2007). Genetics of Parkinson disease.. Genet Med.

[7] Klein C, Pramstaller PP, Kis B, Page CC, Kann M (2000). Parkin deletions in a family with adult-onset, tremor-dominant parkinsonism: expanding the phenotype.. Ann Neurol.

[8] Farrer M, Chan P, Chen R, Tan L, Lincoln S (2001). Lewy bodies and parkinsonism in families with parkin mutations.. Ann Neurol.

[9] Foroud T, Uniacke SK, Liu L, Pankratz N, Rudolph A (2003). Heterozygosity for a mutation in the parkin gene leads to later onset Parkinson disease.. Neurology.

[10] Sun M, Latourelle JC, Wooten GF, Lew MF, Klein C (2006). Influence of heterozygosity for parkin mutation on onset age in familial Parkinson disease: the GenePD study.. Arch Neurol.

[11] Pankratz N, Kissell DK, Pauciulo MW, Halter CA, Rudolph A (2009). Parkin dosage mutations have greater pathogenicity in familial PD than simple sequence mutations.. Neurology.

[12] Wang K, Li M, Hadley D, Liu R, Glessner J (2007). PennCNV: an integrated hidden Markov model designed for high-resolution copy number variation detection in whole-genome SNP genotyping data.. Genome Res.

[13] Colella S, Yau C, Taylor JM, Mirza G, Butler H (2007). QuantiSNP: an Objective Bayes Hidden-Markov Model to detect and accurately map copy number variation using SNP genotyping data.. Nucleic Acids Res.

[14] Simon-Sanchez J, Scholz S, Matarin Mdel M, Fung HC, Hernandez D (2008). Genomewide SNP assay reveals mutations underlying Parkinson disease.. Hum Mutat.

[15] Craddock N, Hurles ME, Cardin N, Pearson RD, Plagnol V Genome-wide association study of CNVs in 16,000 cases of eight common diseases and 3,000 shared controls.. Nature.

[16] Pankratz N, Wilk JB, Latourelle JC, Destefano AL, Halter C (2009). Genomewide association study for susceptibility genes contributing to familial Parkinson disease.. Hum Genet.

[17] Gibb WR, Lees AJ (1988). The relevance of the Lewy body to the pathogenesis of idiopathic Parkinson's disease.. J Neurol Neurosurg Psychiatry.

[18] Gunderson KL, Steemers FJ, Ren H, Ng P, Zhou L (2006). Whole-genome genotyping.. Methods Enzymol.

[19] Karamohamed S, Golbe LI, Mark MH, Lazzarini AM, Suchowersky O (2005). Absence of previously reported variants in the SCNA (G88C and G209A), NR4A2 (T291D and T245G) and the DJ-1 (T497C) genes in familial Parkinson's disease from the GenePD study.. Mov Disord.

[20] Nichols WC, Elsaesser VE, Pankratz N, Pauciulo MW, Marek DK (2007). LRRK2 mutation analysis in Parkinson disease families with evidence of linkage to PARK8.. Neurology.

[21] Nichols WC, Uniacke SK, Pankratz N, Reed T, Simon DK (2004). Evaluation of the role of Nurr1 in a large sample of familial Parkinson's disease.. Mov Disord.

[22] Pankratz N, Pauciulo MW, Elsaesser VE, Marek DK, Halter CA (2006). Mutations in LRRK2 other than G2019S are rare in a north American-based sample of familial Parkinson's disease.. Mov Disord.

[23] Pankratz N, Pauciulo MW, Elsaesser VE, Marek DK, Halter CA (2006). Mutations in DJ-1 are rare in familial Parkinson disease.. Neurosci Lett.

[24] Pankratz N, Nichols WC, Elsaesser VE, Pauciulo MW, Marek DK (2009). Alpha-synuclein and familial Parkinson's disease.. Mov Disord.

[25] Purcell S, Neale B, Todd-Brown K, Thomas L, Ferreira MA (2007). PLINK: a tool set for whole-genome association and population-based linkage analyses.. Am J Hum Genet.

