# Supplementary figures and images for: Copy Number Variation in Familial Parkinson Disease
Source: PLoS One. 2011 Aug 2;6(8):e20988. doi: 10.1371/journal.pone.0020988 (PMC3149037; doi:10.1371/journal.pone.0020988)

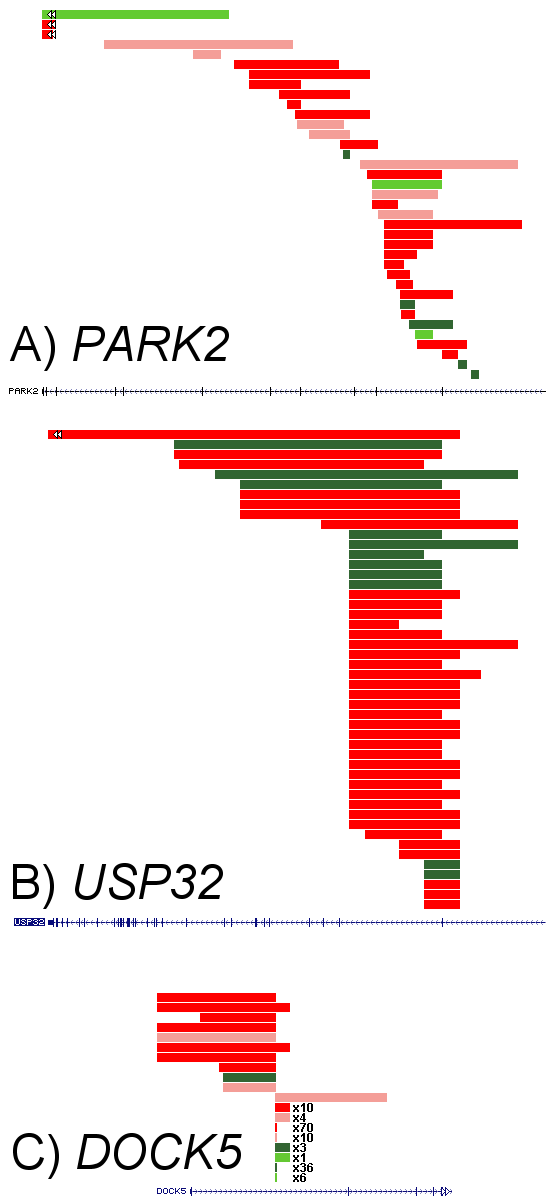

Supplement: Figure S1 — Visualization of CNVs within PARK2 (A), USP32 (B), and DOCK5 (C). Red = Deletion in a case; Pink = Duplication in a case; Dark green = Deletion in a control; Light Green = Duplication in a control; Red followed by ×10 means that ten cases harbored a deletion with the exact same breakpoints; all CNVs displayed in Panel C overlap the same six monomorphic CNV probes (the smallest CNV, deleted ×70 in cases and ×36 in controls); the small arrows in the gene figure indicate direction, the large arrows indicate that not all of the gene is displayed, and the bars indicate exons. (TIF) [file pone.0020988.s001.tif]
