# Supplementary material for: Copy Number Variation in Familial Parkinson Disease
Source: PLoS One. 2011 Aug 2;6(8):e20988. doi: 10.1371/journal.pone.0020988 (PMC3149037; doi:10.1371/journal.pone.0020988)
Supplement: Figure S2 — Sequence for the DOCK5 region and for the primers for the probes in that region. (DOC) [file pone.0020988.s002.doc]

**DOCK5 Intron Sequence**

Primers for PCR / gel electrophoresis

Repeated Sequences:

Form A tacaatatgtatatatgtatacaatatgtgca – 13 repeats

Form B tacaatatgtatagatgtatacaatatgtgca – 2 repeats

Form C tacaatatgtatagatgtatacaatatgtgta – 13 repeats

acaacatgtatatacgtgtatacaatatgtatatatgtatacaatatgtctatacgtgtatacaacatatgtacacaatgtctatatgtgtatacaatatgtatgtatacaatatgtctatacgtgtatacaatatgtatatatgtatacaatatgtgcatacaatatgtatatatgtatacaatatgtgcatacaatatgtatatatgtatacaatatgtgcatacaatatgtatatatgtatacaatatgtgcatacaatatgtatatatgtatacaatatgtgcatacaatatgtatatatgtatacaatatgtgcatacaatatgtatatatgtatacaatatgtgcatacaatatgtatatatgtatacaatatgtgcatacaatatgtatatatgtatacaatatgtgcatacaatatgtatatatgtatacaatatgtgcatacaatatgtatatatgtatacaatatgtgcatacaatatgtatatatgtatacaatatgtgcatacaatatgtatatatgtatacaatatgtgcatacaatatgtatagatgtatacaatatgtgcatacaatatgtatagatgtatacaatatgtgcatacaatatgtatagatgtatacaatatgtgtatacaatatgtatagatgtatacaatatgtgtatacaatatgtatagatgtatacaatatgtgtatacaatatgtatagatgtatacaatatgtgtatacaatatgtatagatgtatacaatatgtgtatacaatatgtatagatgtatacaatatgtgtatacaatatgtatagatgtatacaatatgtgtatacaatatgtatagatgtatacaatatgtgtatacaatatgtatagatgtatacaatatgtgtatacaatatgtatagatgtatacaatatgtgtatacaatatgtatagatgtatacaatatgtgtatacaatatgtatagatgtatacaatatgtgtatacaatatgtatagatgtatacaatatgtgtatacatgtatagatgtatacaatatgtatatatgtatatataatatgtgtatatatacacatatatacgtatatatgtgtatatatacacgaatgcaggagaaggtgatagcattgttcactttcctgttgtagggagagagagagatatatatatatatacacatacacatgtatacatacaatatgtatat

Marker 1: cnv12004PP1

atacaatatgtatatatgtatacaatatgtgcatacaatatgtatatatgtatacaatatgtgcatacaatatgtatatatgtatacaatatgtgcatacaatatgtatat

Marker 2: cnv12004p1

atgtatacaatatgtgcatacaatatgtatatatgtatacaatatgtgcataca

Marker 3: cnv12004PP4

tatatatgtatacaatatgtgcatacaatatgtatatatgtatacaatatgtgcatacaatatgtatatatgtatacaatatgtgcatacaatatgtata

Marker 4: cnv12005p1

acaatatgtgtatacaatatgtatagatgtatacaatatgtgtatacaatatgtatagatgtatacaatatgtgtatacaatatgtatagatgtatacaatatgtgtatac

Marker 5: cnv12005p3

tgtatacaatatgtgtatacaatatgtatagatgtatacaatatgtgtatacaatatgtatagatgtatacaatatgtgtatacaatatgtatagatgtataca

Marker 6: cnv12005p2

caatatgtatagatgtatacaatatgtgtatacaatatgtatagatgtatacaatatgtgtatacaatatgtata
