# Supplementary material for: Copy Number Variation in Familial Parkinson Disease
Source: PLoS One. 2011 Aug 2;6(8):e20988. doi: 10.1371/journal.pone.0020988 (PMC3149037; doi:10.1371/journal.pone.0020988)
Supplement: Table S1 — Comparison of genome-wide results across CNV filters for regions with an empirical genome-wide p-value <0.20 for any test. (DOC) [file pone.0020988.s003.doc]

**Table S1: Comparison of genome-wide results across CNV filters for regions with an empirical genome-wide p-value <0.20 for any test**

|  |  | PennCNV | | | |  | QuantiSNP | | |
| --- | --- | --- | --- | --- | --- | --- | --- | --- | --- |
| Location | Test | Conservative | Common | Union | Gene-centric | Conservative | Common | Union | Gene-centric |
| chr1:173049146-173078950 | window | 1 | 0.13 | 0.13 | 0.07 | 1 | 1 | 1 | 1 |
| chr1:173061043-173067547 | position | 1 | 0.16 | 0.17 | 0.1 | 1 | 1 | 1 | 1 |
| chr3:164101580-164101580 | position | 1 | 1 | 1 | 1 | 1 | 0.18 | 0.18 | 1 |
| chr4:71515698-71763803 | window | 1 | 1 | 1 | 1 | 1 | 0.02 | 0.02 | 0.01 |
| chr4:71528873-71716513 | position | 1 | 1 | 1 | 1 | 1 | 0.18 | 0.17 | 0.12 |
| chr5:151306772-151603385 | window | 1 | 0.01 | 0.02 | 1 | 1 | 0.39 | 0.39 | 1 |
| chr5:151497266-151499003 | position | 1 | 0.02 | 0.02 | 1 | 1 | 0.65 | 0.65 | 1 |
| chr6:162460319-162767020 | window | 0.007 | 0.04 | 0.04 | 0.02 | 0.16 | 0.13 | 0.13 | 0.08 |
| chr8:7141827-7575048 | window | 1 | 0.23 | 0.23 | 0.13 | 0.12 | 1 | 1 | 0.99 |
| chr8:7318603-7356405 | position | 1 | 1 | 1 | 1 | 0.17 | 1 | 1 | 1 |
| chr8:24931867-25191542 | window | 1 | 8.0E-05 | 0.0001 | 0.0001 | 1 | 0.0001 | 0.0001 | 0.0001 |
| chr8:25081094-25135419 | position | 1 | 0.0001 | 0.0001 | 6.0E-05 | 1 | 0.0001 | 0.0001 | 0.0001 |
| chr9:22488756-22492851 | position | 1 | 0.06 | 0.07 | 1 | 1 | 1 | 1 | 1 |
| chr9:44683091-44719629 | position | 1 | 1 | 1 | 1 | 1 | 0.28 | 0.28 | 0.2 |
| chr11:84648923-84988435 | window | 1 | 0.2 | 0.2 | 0.11 | 1 | 0.01 | 0.02 | 0.009 |
| chr11:84731702-84840670 | position | 1 | 0.98 | 0.98 | 0.88 | 1 | 0.04 | 0.04 | 0.03 |
| chr12:36144166-36473504 | window | 1 | 1 | 1 | 1 | 0.14 | 0.97 | 0.97 | 1 |
| chr12:36330311-36407203 | position | 1 | 1 | 1 | 1 | 0.16 | 0.91 | 0.92 | 1 |
| chr14:59991027-59999059 | position | 1 | 1 | 1 | 1 | 1 | 0.27 | 0.27 | 0.19 |
| chr17:55413579-55855046 | window | 1 | 0.0009 | 0.0006 | 0.0005 | 0.001 | 0.001 | 0.001 | 0.0003 |
| chr17:55594073-55785079 | position | 1 | 0.0007 | 0.0006 | 0.0005 | 0.003 | 0.0003 | 0.0007 | 0.0002 |
