# Supplementary material for: Copy Number Variation in Familial Parkinson Disease
Source: PLoS One. 2011 Aug 2;6(8):e20988. doi: 10.1371/journal.pone.0020988 (PMC3149037; doi:10.1371/journal.pone.0020988)
Supplement: Text S1 — Additional Methods and Results. (DOC) [file pone.0020988.s004.doc]

**Supplemental Material**

*Microarray reclustering*

Detection of CNVs utilized the Log R ratio (LRR) and B allele frequency (BAF) measures computed from the signal intensity files using Illumina’s BeadStudio software. These values are generated by converting the normalizing probe intensities X and Y first to R and Theta, where R is the total intensity (X+Y) and Theta is the angle of (X, Y) from the origin (arctangent [Y/X]). R ratio is obtained by comparing the normalized intensity of a subject to that of a reference sample population, after which LRR [log_2_ (R_subject_/R_expected_)] can then be computed for each marker in the dataset. Regions of lower LRR indicate a deletion and regions of higher LRR indicate a duplication. BAF is a function of Theta and estimates the proportion of alleles that are the B allele. BAF values near 0 indicate that the individual harbors no B alleles. Values near 1 indicate that all alleles for that marker are B alleles, and values near 0.5 typically indicate that the individual carries one A allele and one B allele. A deletion will manifest as a series of consecutive markers without BAFs near 0.5 (all at either 0 or 1). A duplication will manifest as a series of markers with BAFs at 0, 0.33, 0.66 and 1 (See Figure 2A).

To ensure the highest quality CNV calls, data were re-clustered (LRRs and BAFs recomputed) using only the white samples that passed quality assessment for the GWAS and using new reference values (R_expected_) generated with the 1,611 (of 1,783) samples that had high quality intensity data (standard deviation of autosomal LRRs < 0.25). QC metrics that would typically be used to filter SNPs for a GWAS (low call rate, low minor allele frequency, deviation from Hardy-Weinberg equilibrium) were not employed to filter markers before CNV detection, as these metrics would likely exclude markers with genuine copy number variation.

*CNV calling algorithms and additional sample filtering criteria*

CNV calls were generated with PennCNV (2008Nov19 version) using the standard hg18 "all" PennCNV hidden Markov model (hmm) and population frequency of B allele (pfb) files. Only variants for which the difference between the log likelihood of the most likely copy number state and the least likely copy number state exceeded 10 (computed using the -conf option in PennCNV) were utilized in subsequent association analyses. PennCNV's default QC parameters were used to assess the quality of each sample’s signal intensities. Any sample with LRR standard deviation >0.32, BAF drift >0.002, waviness factor >0.04 or <-0.04 was removed. Samples where the total number of CNV calls with a confidence value (-conf) >10 was beyond 4 standard deviations from the mean (>95 high-confidence calls) were also removed from further analyses. Using the internal wave adjustment procedure (-gcmodel argument) , the percentage of samples flagged as unusable was reduced from 8.9% to 4.6%. The final sample included 816 cases and 856 controls.

QuantiSNP (Windows v1.1a and Linux64 v1.1b) was also used to call CNVs in the same sample used for PennCNV (816 cases, 856 controls). Analogous CNV filtering steps to those used with PennCNV were performed, resulting in the removal of CNVs that had a Log Bayes Factor value (similar to the PennCNV conf value) smaller than 10. Results from the Windows and Linux versions of QuantiSNP were similar but not exact. Results from the Windows version are presented.

*Visualizing regions of known instability and false positives*

The difference in the DNA source (whole blood versus LCL) can result in spurious association, as is hypothesized for the *DOCK5* locus. The T cell receptor alpha (*TCRA)* gene is one well-known example of a locus that is spontaneously deleted in T-cells, which are a large source of the DNA extracted from whole blood. Therefore it is not surprising that PennCNV called several deletions within this gene for cases, but not for controls, which were all from LCLs. It is for this reason that we and other investigators have excluded this and five other such regions from analysis. When the raw data are viewed in software such as BeadStudio or CNVis, it is apparent that the deletions in *TCRA* are mosaic because they contain heterozygous genotype calls and BAFs between 0.15 and 0.85. For the deletions in *USP32* and *DOCK5*, we did not see such patterns.

*Global Tests of CNVs*

One additional hypothesis was that CNVs would be more prevalent in PD cases as compared with controls. To test this hypothesis, we used Student’s t-test to compare several CNV metrics (total number of CNVs/individual; total summed lengths of the CNVs/individual; average length of each CNV/individual) between cases and controls. More CNV calls were made for cases, particularly CNV calls within genes (p<0.00001 for PennCNV calls), than for controls; but the CNV calls did not tend to be larger CNVs, on average (Table A below). Given that the findings in *USP32* and *DOCK5* were found to be spurious, it is possible that the artifact seen between DNA sources may be more of an issue for probes in and around coding regions. Alternatively, if the association is real, it is noteworthy in so much that the association between PD and the number of CNV calls is greater for the Gene-centric filter (p<0.000001 for the PennCNV calls, which demonstrated a 15% increase) than for the more inclusive filter (p=0.02; 5% increase), and yet no additional genes were nominated by the Gene-centric approach. This would imply that the increased CNV load may be distributed across many different genes, for instance the single *SNCA* duplication mentioned below. Even after the CNVs in *PARK2*, *USP32*, and *DOCK5* are excluded, the association between PD and the number of CNV calls is still significant for the Gene-centric filter (p=0.0003; 12% increase), but not for the more inclusive filter (p=0.06; 4% increase). The average number of CNV calls that did not overlap a gene was slightly smaller in cases versus controls (0.4% decrease).

*Additional Regions identified using the Conservative approach*

The Conservative approach (only analyzing CNVs spanning >100 kb and ≥20 markers) using only PennCNV calls had the highest power to detect *PARK2*, a true positive, and had the lowest false-positive rate. However, as is seen in Table S1, no other region besides *PARK2* came close to genome-wide significance due to the limited power to detect significant differences for rare variants. There were several regions that were unique to cases but that were not statistically significant, including hundreds of regions where a Conservative CNV was seen in a single case and not in a control. Those regions where two or more cases and no controls harbored a Conservative CNV (see Table B below) were the focus of additional examination. Manual review of the LRR and BAF plots revealed that most of these appeared to be spurious calls. As an example, *DACH1* had the most promising CNV count anywhere outside of *PARK2*, but the CNV segments had neither a distinct change in intensity nor the BAF pattern of a deletion or a duplication. This left only *MACROD2* and a handful of regions that had exactly 2 distinct CNVs in cases and none in controls. The *MACROD2* region contained several loci that varied in copy number in 5 cases and 0 controls; however, closer inspection of these CNVs revealed that these 5 CNVs extended to regions of *MACROD2* that were seen in additional cases and a pair of controls. The CNVs (8 cases and 2 controls) were all within a 400 kb window that was entirely intronic. Only two transcripts for *MACROD2* are listed in NCBI’s Gene database, but others are included in UCSC Genome browser (see Supplemental Figure #). The primary *MACROD2* transcript (NM_080676.5; transcript variant 1) defines this region as between exon 5 and exon 7. Exon 6 is not included in this transcript, but in those individuals harboring these CNVs, exon 6 would be deleted in any transcript that would contain it. The other transcript (NM_001033087.1; transcript variant 2) begins with exon 7, making these deletions just 5’ of the transcript. SNP variants in *MACROD2* have been linked with autism (Anney et al. 2010; PMID: 20663923) and brain infarcts (Debette et al 2010; PMID: 20044523).

**References for Supplemental Material**

1. Diskin SJ, Li M, Hou C, Yang S, Glessner J, et al. (2008) Adjustment of genomic waves in signal intensities from whole-genome SNP genotyping platforms. Nucleic Acids Res 36: e126.

2. Walsh T, McClellan JM, McCarthy SE, Addington AM, Pierce SB, et al. (2008) Rare structural variants disrupt multiple genes in neurodevelopmental pathways in schizophrenia. Science 320: 539-543.

**Table A: Comparison of global CNV burden** across filters

|  |  | PennCNV | | | | QuantiSNP | | | |
| --- | --- | --- | --- | --- | --- | --- | --- | --- | --- |
| Test |  | Conservative | Common | Union | Gene-centric | Conservative | Common | Union | Gene-centric |
| Average number | cases | 1.4 CNVs | 12.3 CNVs | 12.5 CNVs | 5.6 CNVs | 3.9 CNVs | 17.3 CNVs | 17.5 CNVs | 9.4 CNVs |
|  | controls | 1.3 CNVs | 11.7 CNVs | 11.9 CNVs | 4.9 CNVs | 3.7 CNVs | 16.8 CNVs | 16.9 CNVs | 8.6 CNVs |
|  | p-value | 0.11 | 0.03 | 0.02 | <0.00001 | 0.13 | 0.17 | 0.17 | 0.01 |
| Summed length | cases | 459 kb | 975 kb | 1036 kb | 739 kb | 1944 kb | 2791 kb | 2840 kb | 2416 kb |
|  | controls | 411 kb | 895 kb | 949 kb | 642 kb | 1826 kb | 2621 kb | 2663 kb | 2205 kb |
|  | p-value | 0.05 | 0.02 | 0.02 | 0.00 | 0.11 | 0.09 | 0.09 | 0.03 |
| Average length | cases | 219 kb | 80 kb | 84 kb | 135 kb | 503 kb | 161 kb | 163 kb | 267 kb |
|  | controls | 224 kb | 76 kb | 79 kb | 132 kb | 504 kb | 155 kb | 156 kb | 269 kb |
|  | p-value | 0.60 | 0.05 | 0.07 | 0.45 | 0.50 | 0.11 | 0.10 | 0.58 |

**Table B:** Regions with Conservative PennCNV calls in cases but not in controls

| Narrow region | Gene | Number  of Cases/  Controls | Nominal/  genome-wide  p-values | Wider region | Number  of Cases/  Controls | Nominal/  genome-wide  p-values | Convincing^1^ |
| --- | --- | --- | --- | --- | --- | --- | --- |
| chr2:106118736-108016226 | several | 2 / 0 | 0.24 / 1.00 | chr2:106118736-108016226 | 2 / 0 | 0.24 / 1.00 | Yes |
| chr5:99026225-99203641 | unknown | 2 / 0 | 0.23 / 1.00 | chr5:98969026-99207162 | 2 / 0 | 0.23 / 1.00 | No |
| chr6:162388114-162552161 | *PARK2* | 5 / 0 | 0.03 / 0.84 | chr2:162250623-162903834 | 15 / 1 | 0.001 / 0.007 | Yes |
| chr9:6658819-6946293 | *JMJD2C* | 2 / 0 | 0.23 / 1.00 | chr9:6680347-7374318 | 3 / 1 | 0.28 / 1.00 | Mixed |
| chr10:96429088-96607307 | *CYP2C19* | 2 / 0 | 0.24 / 1.00 | chr10:96409205-96660698 | 2 / 1 | 0.48 / 1.00 | Yes |
| chr13:41284664-41500335 | *KIAA0564* | 2 / 0 | 0.23 / 1.00 | chr13:41284664-41500335 | 2 / 0 | 0.23 / 1.00 | Yes |
| chr13:70929461-71210958 | *DACH1* | 5 / 0 | 0.03 / 0.84 | chr13:70929461-71210958 | 5 / 0 | 0.03 / 0.84 | No |
| chr16:79736099-79920951 | *BCMO1* | 4 / 0 | 0.06 / 0.99 | chr16:79736099-79920951 | 4 / 1 | 0.18 / 1.00 | Mixed |
| chr19:56972447-56988627 | *FPR3* | 2 / 0 | 0.24 / 1.00 | chr19:56972447-57304917 | 2 / 1 | 0.48 / 1.00 | Yes |
| chr20:5412732-5558738 | *GPCPD1* | 2 / 0 | 0.16 / 1.00 | chr20:5399915-5735786 | 2 / 0 | 0.24 / 1.00 | Yes |
| chr20:14662457-14706111 | *MACROD2* | 3 / 0 | 0.12 / 1.00 | chr20:14625788-15089959 | 8 / 2 | 0.047 / 0.98 | Yes |
| chr20:29401723-29648528 | several | 2 / 0 | 0.24 / 1.00 | chr20:29297270-30059043 | 2 / 0 | 0.24 / 1.00 | Yes |

^1^ This column indicates whether or not the plots of LRR and BAF corroborate the CNV call. For example the probes within the *DACH1* calls were all homozygous genotypes (i.e. no BAF values near 0.50), but the LRR values did not deviate from the rest of the chromosome. Conversely, the *MACROD2* calls had distinct deletion breakpoints and distinct drop in LRR throughout the call.
